# Factors associated with HIV testing among young women in Tanzania: Insights from the 2022 Tanzanian Demographic and Health Survey using Anderson’s Behavioral Model

**DOI:** 10.3389/fpubh.2024.1518314

**Published:** 2025-01-08

**Authors:** Mesfin Abebe, Yordanos Sisay Asgedom, Amanuel Yosef Gebrekidan, Yohannes Addisu Wondimagegne, Habtamu Endashaw Hareru, Tsion Mulat Tebeje

**Affiliations:** ^1^Department of Midwifery, College of Health Sciences and Medicine, Dilla University, Dilla, Ethiopia; ^2^Department of Epidemiology and Biostatistics, College of Health Sciences and Medicine, Wolaita Sodo University, Wolaita Sodo, Ethiopia; ^3^School of Public Health, College of Health Sciences and Medicine, Wolaita Sodo University, Wolaita Sodo, Ethiopia; ^4^School of Public Health, College of Health Sciences and Medicine, Dilla University, Dilla, Ethiopia

**Keywords:** HIV testing, young women, associated factors, Anderson’s Behavioral Model, Tanzania

## Abstract

**Background:**

HIV continues to be a significant global health issue, particularly affecting sub-Saharan Africa, including Tanzania. Knowing one’s HIV status is a crucial first step in combating HIV/AIDS and achieving the targets set for the Sustainable Development Goals (SDGs) by 2030. However, despite ongoing efforts, HIV testing coverage remains low in developing countries, including Tanzania, where testing among young people poses particular challenges. Therefore, this study, based on the 2022 Tanzanian Demographic and Health Survey, aims to identify the factors influencing HIV testing among young women through the lens of Anderson’s Behavioral Model.

**Methods:**

In this study, we analyzed a weighted sample of 5,810 young women from the 2022 Tanzania Demographic and Health Survey (TDHS). Given the hierarchical structure of the DHS data and the binary nature of the outcome variable ‘ever tested for HIV,’ we employed a multilevel mixed-effect logistic regression model. The best-fitting model was identified using the deviance value. In the multivariable analysis, we calculated adjusted odds ratios (AORs) along with their 95% confidence intervals (CIs) to assess the strength of associations between various predictors and HIV testing. Statistical significance was determined at a *p*-value of less than 0.05.

**Result:**

In our study, the prevalence of HIV testing among young women was 60.18% (95% CI: 58.91 to 61.43%). Significant factors associated with HIV testing included age (AOR = 4.33, 95% CI [3.43, 5.45]), marital status (AOR = 2.31, 95% CI [1.76, 3.04]), knowledge of HIV prevention (AOR = 1.59, 95% CI [1.23, 2.04]), discriminatory attitudes towards HIV (AOR = 0.74, 95% CI [0.58, 0.95]), visiting healthcare facilities (AOR = 4.80, 95% CI [3.75, 6.14]), media exposure (AOR = 1.44, 95% CI [1.09, 1.90]), internet use (AOR = 1.56, 95% CI [1.02, 2.38]), and ever heard of STIs (AOR = 2.12, 95% CI [1.63, 2.77]).

**Conclusion:**

Our study found that 60.18% of young women in Tanzania have been tested for HIV. Addressing barriers like stigma and improving access to healthcare and information through media and the internet can significantly boost HIV testing rates among young women, aiding the global effort to end the AIDS epidemic by 2030.The Anderson Behavioral Model emphasizes the importance of predisposing, enabling, and need factors in healthcare utilization, which aligns with our findings and underscores the necessity of a multifaceted approach to improve HIV testing rates.

## Introduction

HIV (Human Immunodeficiency Virus) remains a critical global health challenge, affecting millions of individuals worldwide (1, 2). As of 2023, nearly 40 million people were living with HIV globally. Every minute, one person died from AIDS-related causes. Despite significant progress, almost a quarter of people living with HIV in 2023 (9.3 million) were not receiving medical treatment (3). New HIV infections remained a concern, with an estimated 1.3 million cases in 2023—more than three times the 2025 target of 370,000 or fewer new infections (4). In 2023, about 5.4 million people were unaware that they were living with HIV globally (5). In 2022, the African region had 25.6 million people living with HIV (PLHIV). This region accounts for about 60% of the global new HIV infections. Despite the overall burden of HIV, women experienced a higher prevalence compared to men (6). Women aged 15 to 24 face a heightened risk of contracting HIV, accounting for approximately 26% of new cases worldwide (7). Low and middle-income nations (LMICs), especially those in sub-Saharan Africa (SSA), bear the brunt of the HIV epidemic (8).

Every week in 2023, 4,000 adolescent girls and young women aged 15–24 became infected with HIV globally, with 3,100 of these infections occurring in sub-Saharan Africa (5). Sub-Saharan Africa (SSA) is home to two-thirds of all people living with HIV globally, making it the hardest-hit region (9). Women and girls in SSA accounted for 62% of all new HIV infections, emphasizing the gender disparity (5). In 2023, nearly half of the new HIV infections occurred in eastern and southern Africa, as well as western and central Africa (4). Despite progress in increasing access to HIV testing and treatment services, approximately 9.3 million people living with HIV remained untreated, with 4.7 million of them in sub-Saharan Africa (4).

Although Tanzania has a lower incidence of new HIV infections compared to other sub-Saharan African countries like Lesotho, Eswatini, and South Africa, it still faces severe challenges due to its substantial population of people living with HIV and ongoing social and healthcare barriers (10–12). Among Tanzanian adults, HIV prevalence is 4.4%, with higher rates among women (5.6%) than men (3.0%) (13). Adolescent girls and young women (AGYW) aged 15–24 face barriers such as limited access to sexual and reproductive health services, stigma, lack of education, and socio-economic challenges, impacting their vulnerability to HIV (6, 14). The prevalence of HIV among young people aged 15–19 was 1% (1.3% among girls and 0.8% among boys). Among women aged 20–24, the infection rate was higher (4.4%) than among men (1.7%) (15).

In many African countries, HIV testing coverage varies significantly, ranging from 33.5 to 82.3% (16–20). Voluntary counseling and testing (VCT) play a crucial role as the initial step in detecting, treating, and preventing HIV/AIDS (19). The World Health Organization (WHO) recommends HIV testing and counseling for all patients showing signs and symptoms of the disease, emphasizing its importance in HIV prevention, treatment, care, and support (20, 21). Studies conducted worldwide have identified several factors significantly associated with HIV testing. These factors include demographic characteristics such as (age, gender, and education level), as well as socioeconomic factors like (income, employment status, and access to healthcare services), Behavioral Factors (Having multiple sexual partnerships, recent sexual activity, and history of risky sexual behaviors), Health Knowledge and Awareness (Awareness of HIV/AIDS, media exposure, and knowledge about prevention), and Sexual Practices (condoms utilization during sexual intercourse) (2, 7, 19, 20, 22).

Tanzania aims to end HIV/AIDS as a public health threat by 2030. The 95–95-95 fast-track targets focus on ensuring that 95% of PLHIV know their HIV-positive status, receive treatment, and achieve viral suppression by 2030 (6, 22, 23). Despite interventions, women’s access to and utilization of HIV counseling and testing services remain low, especially among adolescent girls and reproductive-age women (20). Tanzanian women are disproportionately affected, with an HIV prevalence of 6.3% compared to 3.9% among men (15).

Scale-up of HIV testing services has improved awareness, but challenges persist (24). Eastern and southern Africa, including Tanzania, faces slow progress toward the global target of 95% of people knowing their HIV status (24). In 2019, Tanzania introduced HIV testing guidelines, recommending provider-initiated testing and community-based testing (15). Despite efforts, a significant proportion of PLHIV remains undiagnosed, with approximately 22% of Tanzanian women reporting never having been tested (25). Knowing one’s HIV status remains the critical first step in the fight against HIV/AIDS and achieving the 2025 targets (7, 26). Despite efforts, HIV testing coverage remains low in developing countries, including Tanzania (19). Understanding the factors influencing HIV testing behaviors is crucial for developing targeted interventions. Research shows that theory-based approaches are vital for predicting human behaviors (27). This study employs Anderson’s Behavioral Model to analyze the determinants of HIV testing among young women in Tanzania. Anderson’s Behavioral Model posits that health service utilization is influenced by predisposing factors, enabling factors, and need factors (28). To the best of our knowledge, no studies have been conducted on HIV testing among young women in Tanzania using Anderson’s Behavioral Model. This study aims to investigate the prevalence of HIV testing and identify key determinants by analyzing data from the 2022 Tanzania Demographic Health Survey (TDHS) through the lens of Anderson’s Behavioral Model.

## Methods

### Data source

The primary data source for this study was the 2022 Tanzania Demographic Health Survey (TDHS) dataset which was collected cross-sectionally. This dataset includes information collected through household interviews on various health-related topics, such as reproductive health, HIV knowledge, and testing behavior. We extracted relevant variables related to HIV testing from the survey. The 2022 TDHS collected data from a nationally representative probability sample of households, including women of reproductive age and men in the sampled households. This comprehensive dataset provides valuable insights into the health status and behaviors of the Tanzanian population. A total of 15,254 women of reproductive age (15 to 49 years) participated in the survey (29). For this study, a weighted sample of 5,810 young women were included in the final analysis.

### Study population and sampling

Our study population comprises young women aged 15–24 years residing in Tanzania. The sample design was meticulously planned and executed in two stages to ensure that the estimates were representative of the entire country, including both urban and rural areas. In the first stage, primary sampling units (PSUs) were selected with probabilities proportional to their size within strata. These strata were defined by geographic regions and urban/rural areas to ensure comprehensive coverage. In the second stage, a systematic sampling of households was conducted within the selected clusters. This involved listing all households within each selected PSU and then systematically selecting a predetermined number of households from this list (29).

### Outcome variable

The main outcome variable in this secondary analysis was “Ever been tested for HIV/AIDS.” This was measured in the 2022 TDHS as a binary variable. The response options were coded as 0 for ‘No’ (indicating women who had not been tested for HIV/AIDS) and 1 for ‘Yes’ (indicating women who had been tested for HIV/AIDS). This variable was used in the analysis to assess HIV testing among women aged 15–24 years.

### Independent variables

**Figure 1:**
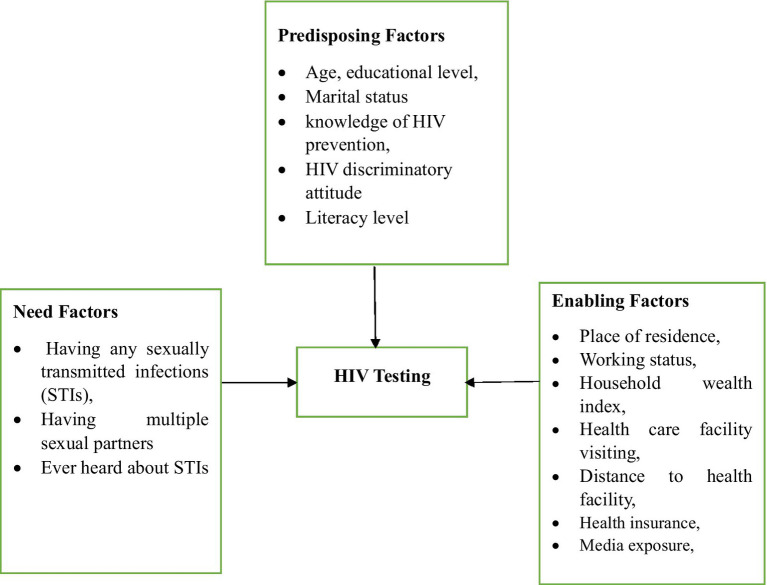
Flowchart of potential influencing factors for hiv testing among young women in tanzania based on adapted Andersen’s Behavioral Model.

By using Andersen’s Behavioral Model and previous literature (27, 30–32), we selected the explanatory variables. In this study, the explanatory variables were categorized into three distinct groups based on Andersen’s Behavioral Model (ABM). According to Andersen’s Behavioral Model (ABM), the categories are predisposing factors, enabling factors, and need-for-care factors (Figure 1). These factors either facilitate or impede an individual’s utilization of health services (27). Predisposing factors include age, educational level, marital status, knowledge of HIV prevention, discriminatory attitudes toward HIV, and literacy level. Enabling factors encompass place of residence, healthcare facility visiting in the last 12 months, distance to healthcare facility, working status, household wealth index, health insurance, and media exposure. The need for care factors include having any sexually transmitted infections (STIs) in the previous 12 months, ever heard of sexually transmitted infections (STIs), and having multiple sexual partners in the previous 12 months (Supporting File).

### Data management and analysis

The data was processed using Stata 17 software, which included extraction, cleaning, recoding, variable transformation and analysis. To ensure the data’s representativeness and obtain accurate estimates and standard errors, weighting was applied before any statistical analysis. Missing data were managed through multiple imputation techniques to handle any gaps and ensure the robustness of our findings. Given the hierarchical nature of the DHS data, a multilevel logistic regression was employed to account for variability among clusters. The Interclass Correlation Coefficient (ICC) was calculated to assess clustering. Both bivariable and multivariable mixed-effects analyses were conducted. Variables with a *p*-value less than 0.2 in the bivariable analysis were included in the multivariable analysis.

### Model building and parameter estimation

In our analysis, we developed four models for the multilevel logistic regression analysis. The first model, the null model, did not include any explanatory variables and served as a baseline. Model II incorporated individual-level factors, while Model III included community-level factors. Model IV combined both individual and community-level factors for a more comprehensive analysis. Model comparison was performed using the deviance statistic −2 log-likelihood (−2LL) to evaluate the fit of four different models. Among these models, Model IV was identified as the best-fitting model due to its lowest deviance value. Multicollinearity was evaluated using the Variance Inflation Factor (VIF), with a cutoff value of 10 (33). The variables “working status” and “multiple sexual partners” both had a VIF of 12.5, leading to their exclusion from the model due to multicollinearity. In the multivariable analysis, variables with a *p*-value of less than 0.05 were considered statistically significant. Finally, the results were presented in both text and table formats.

## Results

### Descriptive characteristics of the study participants

The study included a weighted sample of 5,810 participants, with more than half (53.07%) of the women aged between 15 and 19 years. About 44.47% had a secondary school education, and 76.41% were unmarried. Additionally, 41.71% of participants had knowledge of HIV prevention, while 30.5% held discriminatory attitudes. Furthermore, 64.29% of the young women lived in rural areas, and 44.72% had visited healthcare facilities in the past 12 months. Regarding media exposure, 71.46% of participants were exposed to media, but only 12.89% of young women used the internet. About 71.87% had heard of sexually transmitted infections (STIs), and 13.71% reported a history of STIs (Table 1).

**Table 1:** HIV testing among young women in Tanzania by different background characteristics (*N* = 5,810).

Variables	Category	Weighted frequency (%)	Ever been tested for HIV	
			Yes (%)	No (%)
Age	15–19 years	3,083 (53.07)	1,164 (37.76)	1,919 (62.24)
	20–24 years	2,727 (46.93)	2,333 (85.55)	394 (14.45)
Education level	No education	636 (10.94)	445 (69.97)	191 (30.03)
	Primary	2,550 (43.90)	1,670 (65.49)	880 (34.51)
	Secondary	2,584 (44.47)	1,349 (52.21)	1,235 (47.79)
	Higher	40 (0.69)	32 (80.00)	8 (20.00)
Marital status	Not married	4,440 (76.41)	2,293 (51.64)	2,147 (48.36)
	Married	1,370 (23.59)	1,204 (87.88)	166 (12.12)
knowledge of HIV prevention	No	3,387 (58.29)	1,862 (54.97)	1,525 (45.03)
	Yes	2,423 (41.71)	1,635 (67.48)	788 (32.52)
Discriminatory attitudes towards HIV	No	4,038 (69.50)	2,547 (65.08)	1,491 (36.92)
	Yes	1,772 (30.50)	950 (53.61)	822 (46.39)
Residence	Urban	2,075 (35.71)	1,245 (60.00)	830 (40.00)
	Rural	3,735 (64.29)	2,252 (60.29)	1,483 (39.71)
Healthcare facility visits in the last 12 months	No	3,212 (55.28)	1,396 (43.46)	1,816 (56.54)
	Yes	2,598 (44.72)	2,101 (80.87)	497 (19.13)
Distance to a healthcare facility	Big problem	1,618 (27.85)	972 (60.07)	646 (39.93)
	Not a big problem	4,192 (72.15)	2,525 (60.23)	1,667 (39.77)
Health insurance	No	5,547 (95.48)	3,347 (60.34)	2,200 (39.66)
	Yes	263 (4.52)	150 (57.03)	113 (42.97)
Media exposure	Not Exposed	1,658 (28.54)	946 (57.06)	712 (42.94)
	Exposed	4,152 (71.46)	2,551 (61.44)	1,601 (38.56)
Use of Internet	No	5,061 (87.11)	2,906 (57.42)	2,155 (42.58)
	Yes	749 (12.89)	591 (78.91)	158 (21.09)
Literacy level	Illiterate	854 (14.69)	599 (70.14)	255 (29.86)
	Literate	4,956 (65.31)	2,898 (58.47)	2,058 (41.53)
Wealth index	Poorest	914 (15.74)	547 (59.85)	367 (40.15)
	Poorer	1,005 (17.30)	632 (62.89)	373 (37.11)
	Middle	1,109 (19.08)	676 (60.96)	433 (39.04)
	Richer	1,292 (22.24)	822 (63.62)	470 (36.38)
	Richest	1,490 (25.64)	820 (55.03)	670 (44.97)
Working status	Not working	3,089 (53.16)	1,538 (49.79)	1,551 (50.21)
	Working	2,721 (46.84)	1,959 (72.00)	762 (28.00)
History of any sexually transmitted infections	No	3,214 (86.29)	2,614 (81.33)	600 (18.67)
	Yes	511 (13.71)	461 (90.22)	50 (9.78)
History of multiple sexual partners	No	5,581 (96.07)	3,308 (59.27)	2,273 (40.73)
	Yes	229 (3.93)	189 (82.53)	40 (17.46)
Ever heard of STI	No	1,635 (28.13)	722 (44.16)	913 (55.84)
	Yes	4,175 (71.87)	2,775 (66.47)	1,400 (33.53)

### Prevalence of HIV testing

**Figure 2:**
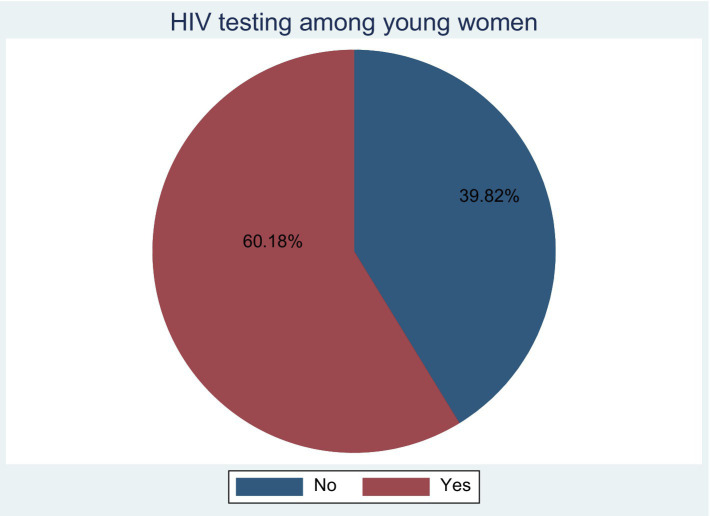
Prevalence of HIV testing among young women in Tanzania.

In this study, the prevalence of HIV testing among young women was 60.18% (95% CI: 58.91%; 61.43%; Figure 2).

### Random effect analysis and model comparison

The Intraclass Correlation Coefficient (ICC) in null model was 8.42% of the variability in HIV testing can be attributed to difference between clusters. Therefore, the multilevel logistic regression model is more suitable for measuring HIV testing uptake (34). The best-performing model was selected based on the lowest deviance value. Model IV, which included both individual and community-level factors, achieved the lowest deviance value (2370.9756) and was identified as the best-fit model (Table 2).

**Table 2:** Multivariable logistic regression analyses of factors associated with HIV testing among young women in Tanzania (*N* = 5,810).

Variables	Null model	Model II AOR (95%CI)	Model III AOR (95%CI)	Model IV AOR (95%CI)
Age				
15–19 years		1		**1**
20–24 years		4.33 (3.44, 5.45)		**4.33 (3.43, 5.45)** ^*^
Education level				
No education		1		1
Primary		1.08 (0.69, 1.70)		1.08 (0.69, 1.70)
Secondary		0.78 (0.47, 1.31)		0.78 (0.47, 1.31)
Higher		0.25 (0.06, 1.01)		0.25 (0.06, 1.01)
Marital status				
Not married		**1**		**1**
Married		2.32 (1.77, 3.04)		**2.31 (1.76, 3.04)** ^*^
Knowledge of HIV prevention				
No		1		**1**
Yes		1.59 (1.23, 2.05)		**1.59 (1.23, 2.04)** ^*^
Discriminatory attitudes towards HIV				
No		1		**1**
Yes		0.74 (0.58, 0.95)		**0.74 (0.58, 0.95)** ^*^
Healthcare facility visits in the last 12 months				
No		1		**1**
Yes		4.80 (3.75, 6.14)		**4.80 (3.75, 6.14)** ^*^
Health insurance				
No		1		1
Yes		1.07 (0.50, 2.27)		1.07 (0.50, 2.27)
Media exposure				
Not Exposed		1		**1**
Exposed		1.45 (1.10, 1.91)		**1.44 (1.09, 1.90)** ^*^
Use of Internet				
No		1		**1**
Yes		1.55 (1.02, 2.37)		**1.56 (1.02, 2.38)** ^*^
Literacy level				
Illiterate		1		1
Literate		0.88 (0.58, 1.34)		0.88 (0.58, 1.35)
Wealth index				
Poorest		1		1
Poorer		1.26 (0.88, 1.80)		1.26 (0.88, 1.80)
Middle		1.40 (0.96, 2.04)		1.41 (0.96, 2.06)
Richer		1.16 (0.78, 1.74)		1.18 (0.77, 1.82)
Richest		0.96 (0.62, 1.49)		0.99 (0.60, 1.64)
History of any sexually transmitted infections				
No		1		1
Yes		1.45 (1.00, 2.11)		1.45 (0.99, 2.11)
Ever heard of STI				
No		1		1
Yes		2.12 (1.63, 2.76)		**2.12 (1.63, 2.77)** ^*^
Distance to a healthcare facility				
Big problem			1	1
Not a big problem			0.95 (0.83, 1.09)	0.99 (0.76, 1.29)
Residence				
Urban			**1**	1
Rural			0.97 (0.84, 1.13)	1.03 (0.73, 1.47)
Model comparison				
LR test	*X*^2^ = 86.20, *p*, <0.0001			
ICC% (95%CI)	8.42 (6.27, 11.21)			
Log-likelihood	−3923.1423	−1185.5129	−3922.9118	−1185.4878
Deviance	7846.2846	2371.0258	7845.8234	2370.9756

^*^p-value < 0.05. Bold values indicate statistically significant variables.

### Factors associated with HIV testing among young women

After conducting bivariate analysis and checking for multicollinearity among the variables using the variance inflation factor, individual and community-level factors of HIV testing among women aged 15–24 years with *p* < 0.2 were selected for multivariable multilevel analysis. These variables included age, educational level, marital status, knowledge of HIV prevention, discriminatory attitudes towards HIV, visits to healthcare facilities in the last 12 months, health insurance, media exposure, internet use, literacy level, wealth index, history of sexually transmitted infections (STIs), ever heard of STIs, distance to healthcare facilities, and residence. In the final model, variables such as age, marital status, knowledge of HIV prevention, discriminatory attitudes towards HIV, visits to healthcare facilities in the last 12 months, media exposure, internet use, and ever heard of STIs were found to be significantly associated with HIV testing among young women.

Women aged 20–24 were 4.33 times more likely to be tested for HIV (AOR = 4.33, 95% CI [3.43, 5.45]) compared to those aged 15–19. Additionally, married women had 2.31 times higher odds of undergoing HIV testing (AOR = 2.31, 95% CI [1.76, 3.04]) compared to their unmarried counterparts. Additionally, women with knowledge of HIV prevention were 59% more likely to be tested for HIV (AOR = 1.59, 95% CI [1.23, 2.04]) than those without such knowledge. The odds of HIV testing were 26% lower among women with discriminatory attitudes towards HIV (AOR = 0.74, 95% CI [0.58, 0.95]) compared to those without such attitudes. Women who visited healthcare facilities in the past 12 months were 4.8 times more likely to be tested for HIV (AOR = 4.80, 95% CI [3.75, 6.14]) compared to those who did not. Additionally, women exposed to media had 44% higher odds of HIV testing (AOR = 1.44, 95% CI [1.09, 1.90]) compared to those without media exposure. Young women who use the internet had 56% higher odds of HIV testing (AOR = 1.56, 95% CI [1.02, 2.38]) compared to those who do not use the internet. Lastly, women who had heard about sexually transmitted infections had more than twice the odds of HIV testing (AOR = 2.12, 95% CI [1.63, 2.77]) compared to those who had not heard about them (Table 2).

## Discussion

This study leverages the 2022 Tanzania Demographic Health Survey (TDHS) to identify the prevalence and factors associated with HIV testing among young age women using Anderson’s Behavioral Model. The study identifies key factors influencing HIV testing in Tanzania, which is essential for informing public health strategies and interventions. In our study, the prevalence of HIV testing among young women was 60.18% (95% CI: 58.91 to 61.43%). This figure is a significant improvement compared to previous studies (22, 35), reflecting the ongoing efforts to enhance HIV awareness and testing services. However, it still falls short of the ambitious targets set by global health initiatives, such as the UNAIDS 95–95-95 targets for 2030, which aim for 95% of people living with HIV to know their status, 95% of those diagnosed to receive sustained antiretroviral therapy, and 95% of those receiving therapy to achieve viral suppression (23, 36).

This finding is higher compared to studies conducted in Sierra Leone (42.1%) (37), Nigeria (23.7%) (38), Burundi (27.1%) (39), Rwanda (55.4%) (40), and Ethiopia (33.5%) (41). It also exceeds the pooled prevalence of HIV testing among young women in Eastern Africa, reported at 55.3% in a multilevel analysis of demographic health survey data (42), and the prevalence in sub-Saharan Africa, which stands at 36.5% (31). Similarly, research in Ghana found that only 31.4% of young women had been tested for HIV (7). In contrast, our finding is lower than the pooled HIV testing prevalence of 64.4% reported in a study across 28 sub-Saharan African countries (43). This finding is also lower than a study done in Malawi (69.5%) (44). These comparisons highlight Tanzania’s progress in promoting HIV testing among young women, which could be attributed to effective public health campaigns and accessible healthcare services. The variation in HIV testing prevalence among young women observed in our study, compared to other regions and countries, can be attributed to several factors. Firstly, the significant improvement in our study area reflects the successful implementation of targeted HIV awareness and testing campaigns, which have likely increased accessibility and acceptance of testing services. Additionally, differences in healthcare infrastructure, availability of resources, and socio-cultural factors across countries can influence testing rates. Furthermore, variations in the timing and methodology of data collection across studies can also contribute to these differences. Overall, while our findings indicate progress, they highlight the need for continued efforts to address barriers to HIV testing and to meet global health targets.

Our study found that women aged 20–24 years had significantly higher odds of being tested for HIV (AOR = 4.33, 95% CI [3.43, 5.45]) compared to those aged 15–19. This aligns with findings from Rwanda (40), Zambia (45), Burundi (39), South Africa (46), Tanzania (47), Sierra Leone (37), and sub-Saharan Africa (31), where older age was associated with higher odds of HIV testing. The increased likelihood of testing among older women may be due to greater sexual activity, higher risk perception, and more frequent interactions with healthcare services. Policymakers should consider age-specific strategies to encourage HIV testing, such as tailored educational campaigns and youth-friendly health services.

Married women in our study had higher odds of HIV testing (AOR = 2.31, 95% CI [1.76, 3.04]) compared to unmarried women. This is consistent with findings from Ghana (7), Zambia (17), and Ethiopia (41) where being married was associated with higher odds of HIV testing. A possible reason for this finding is that marriage often entails more frequent healthcare visits, such as antenatal care, which can increase opportunities for HIV testing. Additionally, there may be greater social and familial support for married women to undergo testing, as well as a perceived responsibility to protect their spouse and future children from HIV. Policies should address these differences by providing support systems that cater to the unique needs of both groups. For instance, unmarried women could be targeted through community outreach programs.

Women with knowledge of HIV prevention were more likely to be tested for HIV (AOR = 1.59, 95% CI [1.23, 2.04]). This finding aligns with studies conducted in Eastern Africa (42), sub-Saharan Africa (31), and South Africa (32), which demonstrates that knowledge about HIV/AIDS prevention significantly increases the likelihood of HIV testing. One possible reason for this finding is that women who are knowledgeable about HIV prevention are more likely to understand the benefits of early detection and treatment. This awareness can lead to proactive health-seeking behaviors, including getting tested for HIV.

Our study revealed that discriminatory attitudes towards HIV significantly reduce the likelihood of HIV testing (AOR = 0.74, 95% CI [0.58, 0.95]). This aligns with research conducted in Nigeria (48), Ethiopia (49), and three other sub-Saharan African countries (50), which also identified stigma as a major obstacle to HIV testing. To enhance testing rates, it is essential to reduce stigma through targeted education and awareness campaigns. In the context of our findings, discriminatory attitudes towards HIV can be seen as a significant predisposing factor. These attitudes are often rooted in societal stigma and misinformation about HIV, which can deter individuals from seeking testing due to fear of judgment or social repercussions (50). This aligns with previous research indicating that stigma and discrimination are major barriers to HIV testing and care (51).

Women who visited healthcare facilities in the last 12 months had significantly higher odds of HIV testing (AOR = 4.80, 95% CI [3.75, 6.14]). This finding is consistent with studies conducted in East Africa (42), and Sierra Leone (37), which highlight the importance of healthcare access in promoting HIV testing. This may be due to provider-initiated testing, where healthcare providers offer HIV testing as part of routine care. This approach increases the likelihood of testing by integrating it into regular health visits, making it more accessible and reducing the stigma associated with seeking out testing independently. Those who engage with healthcare facilities are more likely to be tested for HIV, reflecting the positive impact of provider-initiated efforts on increasing testing uptake among women (52). Another possible reason may be that regular interactions with healthcare providers enhance opportunities for HIV testing and counseling. These interactions build trust and rapport, making women more comfortable discussing sensitive health issues. Healthcare providers can offer personalized advice, address concerns, and provide information about the benefits of HIV testing, encouraging women to get tested (53).

Exposure to media was associated with higher odds of HIV testing in our study (AOR = 1.44, 95% CI [1.09, 1.90]). Similar findings have been reported in other studies (40, 42), where media exposure was linked to increased awareness and uptake of HIV testing. Media campaigns are effective in disseminating crucial information, and encouraging individuals to get tested. Our findings also showed that young age women in Tanzania who use the Internet had higher odds of getting tested for HIV compared to those who did not (AOR = 1.56, 95% CI [1.02, 2.38]). This finding is supported by a study done in Sierra Leone (37). The Internet offers a vast array of information on HIV/AIDS, covering aspects such as transmission, prevention, and testing options. Young age women who use the Internet are more likely to come across educational campaigns or resources that encourage HIV testing (37).

Lastly, women who had heard about sexually transmitted infections (STIs) had more than twice the odds of HIV testing (AOR = 2.12, 95% CI [1.63, 2.77]). Awareness and knowledge about STIs and their link to HIV can significantly influence the uptake of HIV testing. Studies have shown that individuals who are knowledgeable about STIs and HIV are more likely to get tested for both (54). This is because understanding the risks and consequences of untreated STIs can motivate individuals to seek testing and treatment. Awareness of STIs directly correlates with perceived need. Women who know about STIs may perceive themselves at higher risk of HIV, thus feeling a greater need to get tested. This perceived need drives the actual utilization of HIV testing services (55).

### Implication of the study

The findings of this study identify key factors of HIV testing behavior among young women in Tanzania, emphasizing the importance of targeted interventions. With a prevalence of 60.18%, it is evident that while a significant proportion of young women are getting tested, there remains a substantial gap in achieving universal testing. The significant associations with factors such as age, marital status, and knowledge of HIV prevention suggest that educational and awareness programs tailored to different demographic groups could enhance testing rates. Additionally, addressing discriminatory attitudes through community education and advocacy, and improving access to healthcare facilities by increasing funding and resources, are crucial for increasing HIV testing uptake. The role of media exposure and internet use underscores the potential of leveraging digital platforms for health communication. These insights can inform policymakers and healthcare providers in designing comprehensive strategies to improve HIV testing coverage, ultimately contributing to the achievement of global health targets like the UNAIDS 95–95-95 goals (26, 36). To improve HIV testing rates, it is crucial to integrate HIV testing services with other reproductive health services. This approach can reduce the stigma associated with HIV testing and make it more accessible to young women.

### Strengths and limitations of the study

The study utilizes data from the 2022 Tanzanian Demographic and Health Survey, which is a nationally representative dataset. This allows for robust and generalizable findings across different regions and demographics within Tanzania. The use of Anderson’s Behavioral Model also provides a structured framework to understand the multifaceted factors influencing HIV testing behavior among young women. The study’s cross-sectional nature limits the ability to infer causality. While associations between various factors and HIV testing can be identified, it is not possible to determine whether these factors directly cause changes in HIV testing behavior. Self-reported data introduces potential response bias, as participants may provide socially desirable answers, affecting the accuracy of our findings. We also excluded some variables due to multicollinearity, which can distort regression results and complicate the determination of each variable’s individual effect. This exclusion, while necessary for robustness, may have omitted relevant factors.

To validate causality, we recommend conducting longitudinal studies. These studies would allow for the observation of changes over time and provide a clearer understanding of the causal relationships between the variables influencing HIV testing behaviors. Additionally, longitudinal research could help identify trends and long-term effects, offering valuable insights for developing more effective interventions.

## Conclusion

Our study found that 60.18% of young women in Tanzania have been tested for HIV. This study highlights several factors significantly associated with HIV testing among young women, including age, marital status, knowledge of HIV prevention, discriminatory attitudes, healthcare facility visits, media exposure, internet use, and awareness of STIs. These findings align with other studies conducted in sub-Saharan Africa and beyond. Our study underscores the importance of addressing barriers such as stigma and improving access to healthcare and information through media and the internet. Enhancing these areas can significantly boost HIV testing rates among young women, contributing to the global effort to end the AIDS epidemic by 2030. The Anderson Behavioral Model emphasizes the importance of predisposing, enabling, and need factors in healthcare utilization, which aligns with our findings and underscores the necessity of a multifaceted approach to improve HIV testing rates.

## Data Availability

The datasets presented in this study can be found in online repositories. The names of the repository/repositories and accession number(s) can be found below: the dataset is publicly accessible online. Anyone interested can obtain it by visiting the official website at www.measuredhs.com and following the necessary steps to access the data.

## References

[1] Payagala S Pozniak A. The global burden of HIV. Clin Dermatol. (2024) 42:119–27. doi: 10.1016/j.clindermatol.2024.02.001, PMID: 38387533

[2] Joseph F Jean Simon D Kondo Tokpovi VC Kiragu A Toudeka M-RAS Nazaire R. Trends and factors associated with recent HIV testing among women in Haiti: a cross-sectional study using data from nationally representative surveys. BMC Infect Dis. (2024) 24:74. doi: 10.1186/s12879-023-08936-z, PMID: 38212702 PMC10782569

[3] Iacobucci G. UN agency calls for funding boost and political will to end AIDS by 2030. Br Med J. (2024) 386:q1647. doi: 10.1136/bmj.q1647, PMID: 39043415

[4] HIV/AIDS JUNPo (2023). The path that ends AIDS: 2023 UNAIDS global AIDS update.

[5] HIV/AIDS JUNPo (2023). Global HIV & AIDS statistics—Fact sheet. (Accessed August 8, 2024).

[6] Mboya E Mizinduko M Balandya B Mushi J Sabasaba A Amani DE. HIV burden and the global fast-track targets progress among pregnant women in Tanzania calls for intensified case finding: analysis of 2020 antenatal clinics HIV sentinel site surveillance. PLoS One. (2023) 18:e0285962. doi: 10.1371/journal.pone.0285962, PMID: 37824470 PMC10569580

[7] Essuman MA Mohammed H Kebir MS Obiribea C Ahinkorah BO. Prevalence and factors associated with HIV testing among young women in Ghana. BMC Infect Dis. (2024) 24:416. doi: 10.1186/s12879-024-09068-8, PMID: 38641776 PMC11027531

[8] Musuka G Moyo E Cuadros D Herrera H Dzinamarira T. Redefining HIV care: a path toward sustainability post-UNAIDS 95-95-95 targets. Front Public Health. (2023) 11:1273720. doi: 10.3389/fpubh.2023.1273720, PMID: 37927857 PMC10620686

[9] Hailu BA. Trend and principal components of HIV/AIDS among adults in SSA. Sci Rep. (2024) 14:11098. doi: 10.1038/s41598-024-55872-2, PMID: 38750039 PMC11096374

[10] Mkwashapi D Renju J Mahande M Changalucha J Urassa M Todd J. Fertility trends by HIV status in a health and demographic surveillance study in Magu District, Tanzania, 1994–2018. PLoS One. (2023) 18:e0281914. doi: 10.1371/journal.pone.0281914, PMID: 36802408 PMC9942988

[11] Wang Y Jing W Liu J Liu M. Global trends, regional differences and age distribution for the incidence of HIV and tuberculosis co-infection from 1990 to 2019: results from the global burden of disease study 2019. Infect Dis. (2022) 54:773–83. doi: 10.1080/23744235.2022.2092647, PMID: 35801264

[12] Carter A Zhang M Tram KH Walters MK Jahagirdar D Brewer ED. Global, regional, and national burden of HIV/AIDS, 1990–2021, and forecasts to 2050, for 204 countries and territories: the global burden of disease study 2021. Lancet HIV. (2024) 11:e807–22. doi: 10.1016/S2352-3018(24)00212-1, PMID: 39608393 PMC11612058

[13] TNBo S. Tanzania HIV Impact Survey (THIS) 2022–2023. Tanzania National Bureau of Statistics. (2023).

[14] Madut DB Manavalan P Mtalo A Peter T Ostermann J Njau B. Predictors of prior HIV testing and acceptance of a community-based HIV test offer among male bar patrons in northern Tanzania. PLoS Glob Public Health. (2024) 4:e0002946. doi: 10.1371/journal.pgph.0002946, PMID: 38408037 PMC10896543

[15] National AIDS Control Programme (NACP) (2019). National Comprehensive Guidelines on HIV testing Services in Tanzania. 3d ed. National AIDS Control Program 2019. p. e0266870.

[16] Salima N Leah E Stephen L. HIV testing among women of reproductive age exposed to intimate partner violence in Uganda. Open Public Health J. (2018) 11:275–87. doi: 10.2174/1874944501811010275

[17] Muyunda B Mee P Todd J Musonda P Michelo C. Estimating levels of HIV testing coverage and use in prevention of mother-to-child transmission among women of reproductive age in Zambia. Arch Public Health. (2018) 76:1–9. doi: 10.1186/s13690-018-0325-x, PMID: 30619607 PMC6310990

[18] Nall A Chenneville T Rodriguez LM O’Brien JL. Factors affecting HIV testing among youth in Kenya. Int J Environ Res Public Health. (2019) 16:1450. doi: 10.3390/ijerph16081450, PMID: 31022872 PMC6517959

[19] Worku MG Tesema GA Teshale AB. Prevalence and associated factors of HIV testing among reproductive-age women in eastern Africa: multilevel analysis of demographic and health surveys. BMC Public Health. (2021) 21:1262. doi: 10.1186/s12889-021-11292-9, PMID: 34187431 PMC8243417

[20] Deynu M Agyemang K Anokye N. Factors associated with HIV testing among reproductive women aged 15–49 years in the Gambia: analysis of the 2019–2020 Gambian demographic and health survey. Int J Environ Res Public Health. (2022) 19:4860. doi: 10.3390/ijerph19084860, PMID: 35457730 PMC9031325

[21] Zegeye B Adjei NK Ahinkorah BO Tesema GA Ameyaw EK Budu E. HIV testing among women of reproductive age in 28 sub-Saharan African countries: a multilevel modelling. Int Health. (2023) 15:573–84. doi: 10.1093/inthealth/ihad031, PMID: 37099414 PMC10472880

[22] Wang Y Kinsler JJ Kiwuwa-Muyingo S. Factors associated with HIV testing among youth in Tanzania based on the 2016–2017 Tanzania HIV impact survey (THIS). PLoS Glob Public Health. (2022) 2:e0000536. doi: 10.1371/journal.pgph.0000536, PMID: 36589732 PMC9799957

[23] HIV/AIDS JUNPo (2022). Ending inequalities and getting on track to end AIDS by 2030: A summary of the commitments and targets within the United Nations general Assembly’s 2021 political declaration on HIV and AIDS.

[24] Grimsrud A Wilkinson L Ehrenkranz P Behel S Chidarikire T Chisenga T. The future of HIV testing in eastern and southern Africa: broader scope, targeted services. PLoS Med. (2023) 20:e1004182. doi: 10.1371/journal.pmed.1004182, PMID: 36917570 PMC10013883

[25] Martelli G Van Duffel L Kwezi EC Cavallin F Salehe IA Torelli GF. Community-and facility-based HIV testing interventions in northern Tanzania: midterm results of Test & Treat Project. PLoS One. (2022) 17:e0266870. doi: 10.1371/journal.pone.0266870, PMID: 35413074 PMC9004748

[26] Frescura L Godfrey-Faussett P Feizzadeh AA El-Sadr W Syarif O Ghys PD. Achieving the 95 95 95 targets for all: a pathway to ending AIDS. PLoS One. (2022) 17:e0272405. doi: 10.1371/journal.pone.0272405, PMID: 35925943 PMC9352102

[27] Seidu A-A. Using Anderson's model of health service utilization to assess the use of HIV testing services by sexually active men in Ghana. Front Public Health. (2020) 8:512. doi: 10.3389/fpubh.2020.00512, PMID: 33042949 PMC7522213

[28] Andersen R Newman JF. Societal and individual determinants of medical care utilization in the United States. Milbank Q. (2005) 83:Online-only-Online-only. doi: 10.1111/j.1468-0009.2005.00428.x, PMID: 4198894

[29] Ministry of Health (MoH) [Tanzania Mainland], Ministry of Health (MoH) [Zanzibar], National Bureau of Statistics (NBS), Office of the Chief Government Statistician (OCGS), and ICF. (2022). Tanzania demographic and health survey and malaria Indicator survey 2022 final report. Dodoma, Tanzania, and Rockville, Maryland, USA: MoH, NBS, OCGS, and ICF

[30] Sonko I Chung M-H Hou W-H Chen W-T Chang P-C. Predictors of HIV testing among youth aged 15–24 years in the Gambia. PLoS One. (2022) 17:e0263720. doi: 10.1371/journal.pone.0263720, PMID: 35180256 PMC8856544

[31] Asaolu IO Gunn JK Center KE Koss MP Iwelunmor JI Ehiri JE. Predictors of HIV testing among youth in sub-Saharan Africa: a cross-sectional study. PLoS One. (2016) 11:e0164052. doi: 10.1371/journal.pone.0164052, PMID: 27706252 PMC5051677

[32] Peltzer K Matseke G. Determinants of HIV testing among young people aged 18–24 years in South Africa. Afr Health Sci. (2013) 13:1012–20. doi: 10.4314/ahs.v13i4.22, PMID: 24940326 PMC4056506

[33] Midi H Sarkar SK Rana S. Collinearity diagnostics of binary logistic regression model. J Interdis Math. (2010) 13:253–67. doi: 10.1080/09720502.2010.10700699

[34] Kianoush F Masoomehni K. Application REML model and determining cut off of ICC by multi-level model based on Markov chains simulation in health. Indian J Fund Appl Life Sci. (2015) 5:1432–48.

[35] Mahande MJ Phimemon RN Ramadhani HO. Factors associated with changes in uptake of HIV testing among young women (aged 15–24) in Tanzania from 2003 to 2012. Infect Dis Poverty. (2016) 5:64–75. doi: 10.1186/s40249-016-0180-3, PMID: 27595846 PMC5011841

[36] Heath K Levi J Hill A. The Joint United Nations Programme on HIV/AIDS 95–95–95 targets: worldwide clinical and cost benefits of generic manufacture. AIDS. (2021) 35:S197–203. doi: 10.1097/QAD.0000000000002983, PMID: 34115649

[37] Osborne A Bangura C Williams SMT Koroma AH Fornah L Yillah RM. Spatial distribution and factors associated with HIV testing among adolescent girls and young women in Sierra Leone. BMC Infect Dis. (2024) 24:1192. doi: 10.1186/s12879-024-10031-w, PMID: 39438853 PMC11515748

[38] Ajayi AI Awopegba OE Adeagbo OA Ushie BA. Low coverage of HIV testing among adolescents and young adults in Nigeria: implication for achieving the UNAIDS first 95. PLoS One. (2020) 15:e0233368. doi: 10.1371/journal.pone.0233368, PMID: 32428005 PMC7237011

[39] Nshimirimana C Vuylsteke B Smekens T Benova L. HIV testing uptake and determinants among adolescents and young people in Burundi: a cross-sectional analysis of the demographic and health survey 2016–2017. BMJ Open. (2022) 12:e064052. doi: 10.1136/bmjopen-2022-064052, PMID: 36229150 PMC9562752

[40] Musekiwa A Silinda P Bamogo A Twabi HS Mohammed M Batidzirai JM. Prevalence and factors associated with self-reported HIV testing among adolescent girls and young women in Rwanda: evidence from 2019/20 Rwanda demographic and health survey. BMC Public Health. (2022) 22:1281. doi: 10.1186/s12889-022-13679-8, PMID: 35778711 PMC9250268

[41] Bekele YA Fekadu GA. Factors associated with HIV testing among young females; further analysis of the 2016 Ethiopian demographic and health survey data. PLoS One. (2020) 15:e0228783. doi: 10.1371/journal.pone.0228783, PMID: 32045460 PMC7012428

[42] Worku MG Teshale AB Tesema GA. Prevalence and associated factors of HIV testing among young (15–24) women in eastern Africa: a multilevel analysis of demographic health survey data (2008-2018). Arch Public Health. (2022) 80:117. doi: 10.1186/s13690-022-00879-2, PMID: 35410302 PMC9004117

[43] Seidu A-A Oduro JK Ahinkorah BO Budu E Appiah F Baatiema L. Women’s healthcare decision-making capacity and HIV testing in sub-Saharan Africa: a multi-country analysis of demographic and health surveys. BMC Public Health. (2020) 20:1–11. doi: 10.1186/s12889-020-09660-y 33092556 PMC7583279

[44] Koroma M Chigoneka K Kabba J Yu J-R Wang L Xie D-J. Factors influencing HIV testing among young women aged 15-24 according to the 2015-2016 Malawi demographic and health survey. HIV & AIDS review. Int J HIV-Relat Prob. (2023) 22:237–44. doi: 10.5114/hivar.2023.131606, PMID: 39649281

[45] Heri AB Cavallaro FL Ahmed N Musheke MM Matsui M. Changes over time in HIV testing and counselling uptake and associated factors among youth in Zambia: a cross-sectional analysis of demographic and health surveys from 2007 to 2018. BMC Public Health. (2021) 21:1–18. doi: 10.1186/s12889-021-10472-x, PMID: 33676482 PMC7937241

[46] Musekiwa A Bamogo A Shisana O Robsky K Zuma K Zungu NP. Prevalence of self-reported HIV testing and associated factors among adolescent girls and young women in South Africa: results from a 2017 nationally representative population-based HIV survey. Public Health Pract. (2021) 2:100093. doi: 10.1016/j.puhip.2021.100093, PMID: 36101581 PMC9461291

[47] Damian DJ Msuya SE, editors. (2016). HIV prevalence and factors associated with HIV testing among young people (15–24 years) in Tanzania. 2016 annual meeting: PAA.

[48] Ibrahim M Ipadeola O Adebayo S Fatusi A. Socio-demographic determinants of HIV counseling and testing uptake among young people in Nigeria. Int J Prevent Treat. (2013) 2:23–31. doi: 10.5923/j.ijpt.20130203.01

[49] Teklehaimanot HD Teklehaimanot A Yohannes M Biratu D. Factors influencing the uptake of voluntary HIV counseling and testing in rural Ethiopia: a cross sectional study. BMC Public Health. (2016) 16:1–13. doi: 10.1186/s12889-016-2918-z, PMID: 26955869 PMC4784416

[50] Workneh BS Zegeye AF Tamir TT Ali MS Ayenew T Mekonen EG. Individual and community level factors associated with discriminatory attitudes against people living with HIV/AIDS among women of reproductive age in three sub-Saharan African countries: evidence from the most recent demographic and health survey (2021/22). BMC Public Health. (2024) 24:1503. doi: 10.1186/s12889-024-19022-7, PMID: 38840148 PMC11151550

[51] Hempel S Ferguson L Bolshakova M Yagyu S Fu N Motala A. Frameworks, measures, and interventions for HIV-related internalised stigma and stigma in healthcare and laws and policies: systematic review protocol. BMJ Open. (2021) 11:e053608. doi: 10.1136/bmjopen-2021-053608, PMID: 34887280 PMC8663079

[52] Sundararajan R Ponticiello M Nansera D Jeremiah K Muyindike W. Interventions to increase HIV testing uptake in global settings. Curr HIV/AIDS Rep. (2022) 19:184–93. doi: 10.1007/s11904-022-00602-4, PMID: 35441985 PMC9110462

[53] Organization WH. Policy brief: Consolidated guidelines on HIV prevention, diagnosis, treatment and care for key populations. WHO: World Health Organization (2017). 25996019

[54] Fonner VA Armstrong KS Kennedy CE O'Reilly KR Sweat MD. School based sex education and HIV prevention in low-and middle-income countries: a systematic review and meta-analysis. PLoS One. (2014) 9:e89692. doi: 10.1371/journal.pone.0089692, PMID: 24594648 PMC3942389

[55] Shewarega ES Fentie EA Asmamaw DB Negash WD Fetene SM Teklu RE. Sexually transmitted infections related care-seeking behavior and associated factors among reproductive age women in East Africa: a multilevel analysis of demographic and health surveys. BMC Public Health. (2022) 22:1714. doi: 10.1186/s12889-022-14120-w, PMID: 36085047 PMC9463758

