# Supplementary material for: Factors associated with HIV testing among young women in Tanzania: Insights from the 2022 Tanzanian Demographic and Health Survey using Anderson’s Behavioral Model
Source: Front Public Health. 2025 Jan 8;12:1518314. doi: 10.3389/fpubh.2024.1518314 (PMC11750764; doi:10.3389/fpubh.2024.1518314)
Supplement: Supplementary file 1 [file Table_1.DOCX]

Table 1: Detailed Description and Measurement of Independent Variables

| Variable | Description |
| --- | --- |
| Predisposing Factors |  |
| Age | Young women were categorized into two age groups: 15-19 years (coded as “0”) and 20-24 years (coded as “1”). |
| Educational status | Young women were grouped based on their highest level of education: no education (coded as “0”), primary education (coded as “1”), secondary education (coded as “2”), and higher education (coded as “3”). |
| Marital status | Young women were categorized as unmarried (never in union, divorced, widowed, separated) (coded as “0”) or married (living with a partner or married) (coded as “1”). |
| Knowledge of HIV prevention | A young woman is considered to have comprehensive knowledge if she answers all five questions correctly. If she fails to answer even one question correctly, she is not considered knowledgeable. |
| HIV discriminatory attitude | A young woman is considered to have discriminatory attitudes towards people living with HIV if she responds that she would not buy fresh vegetables from a vendor known to have HIV, or if she believes that children with HIV should not be allowed to attend school with children who do not have HIV (Yes or No). |
| Literacy | Young women were classified as either illiterate (coded as “0”) or literate (coded as “1”). |
| **Enabling Factors** |  |
| Place of residence | This variable indicated whether young women lived in rural or urban areas, |
| Healthcare facility visiting | Young women were categorized based on whether they visited a health facility in the last 12 months (coded as “0”) or not (coded as “1”). |
| Distance to a healthcare facility | Young women were classified according to whether they perceived getting medical help as a big problem (coded as “0”) or not a big problem (coded as “1”). |
| Working status | Young women were classified as either not working (coded as “0”) or working (coded as “1”). |
| Household wealth index | The wealth index was originally divided into five categories: poorest, poorer, middle, richer, and richest. For this study, it was simplified into three categories: poor (poorest and poorer), middle, and rich (richer and richest). |
| Health insurance | Health insurance availability |
| Media exposure | This variable combined whether a young woman reads newspapers/magazines, listens to the radio, or watches television. It was coded as “0” if she was not exposed to any of these media and “1” if she was exposed to at least one. |
| Internet used | This variable indicated whether young women used the internet or not. |
| **Need Factors** | |
| History of any sexually transmitted infections (STIs) | Young women were classified based on history of any sexually transmitted infections (STIs) in the past 12 months, with responses coded as “0” for no history of STIs and “1” for a history of STIs. |
| History of multiple sexual partners | Young women were categorized based on the number of sexual partners in the past 12 months: zero or one partner (coded as “0”) and more than one partner (coded as “1”). |
